# Robot-Assisted Maxillary Positioning in Orthognathic Surgery: A Feasibility and Accuracy Evaluation

**DOI:** 10.3390/jcm10122596

**Published:** 2021-06-11

**Authors:** Jeong Joon Han, Sang-Yoon Woo, Won-Jin Yi, Soon Jung Hwang

**Affiliations:** 1 Department of Oral and Maxillofacial Surgery, School of Dentistry, Dental Research Institute, Seoul National University, Seoul 03080, Korea; ooops01@snu.ac.kr; 2 Department of Biomedical Radiation Sciences, Graduate School of Convergence Science and Technology, Seoul National University, Seoul 03080, Korea; woodli14@snu.ac.kr; 3 Department of Oral and Maxillofacial Radiology, School of Dentistry, Dental Research Institute, Seoul National University, Seoul 03080, Korea; 4 Hwang Soon Jung’s Dental Clinic for Oral and Maxillofacial Surgery, Woonam Building, 2,3 F, 349, Gangnam-daero, Seocho-gu, Seoul 06626, Korea

**Keywords:** robot-assisted surgery, maxillary repositioning, orthognathic surgery, accuracy

## Abstract

Several methods enabling independent repositioning of the maxilla have been introduced to reduce intraoperative errors inherent in the intermediate splint. However, the accuracy is still to be improved and a different approach without time-consuming laboratory process is needed, which can allow perioperative modification of unoptimized maxillary position. The purpose of this study is to assess the feasibility and accuracy of a robot arm combined with intraoperative image-guided navigation in orthognathic surgery. The experiments were performed on 12 full skull phantom models. After Le Fort I osteotomy, the maxillary segment was repositioned to a different target position using a robot arm and image-guided navigation and stabilized. Using the navigation and the postoperative computed tomography (CT) images, the achieved maxillary position was compared with the planned position. Although the maxilla showed mild displacement during the fixation, the mean absolute deviations from the target position were 0.16 mm, 0.18 mm, and 0.20 mm in medio-lateral, antero-posterior, and supero-inferior directions, respectively, in the intraoperative navigation. Compared with the target position using postoperative CT, the achieved maxillary position had a mean absolute deviation of less than 0.5 mm for all dimensions and the mean root mean square deviation was 0.79 mm. The results of this study suggest that the robot arm combined with the intraoperative image-guided navigation may have great potential for surgical plan transfer with the accurate repositioning of the maxilla in the orthognathic surgery.

## 1. Introduction

Orthognathic surgery has been widely used as a successful treatment for dentofacial deformities [1,2]. One of the factors that have the greatest influence on the treatment outcome and patient satisfaction after orthognathic surgery is the accuracy of the surgery. To transfer the surgical plan to the operation theater, the repositioning of the maxillary segment using the autorotation of the maxillomandibular complex has been most widely used in the past [3]. However, the traditional repositioning method using intermediate splint requires several error-prone laboratory procedures and has an inherent problem of inaccurate positioning in vertical positioning [4,5].

To replace this method, several approaches such as intraoperative navigation, template-based maxillary repositioning, and patient-specific implants (PSI) have been introduced [6,7,8]. These methods enable independent repositioning of the maxilla without the help of mandibular autorotation, therefore, the possibility of an intraoperative error inherent in the intermediate splint can be reduced [9,10] and there is no need for error-prone and time-consuming laboratory procedures for fabrication of the intermediate splint. Even though various attempts have been made for orthognathic surgery using intraoperative navigation [11,12,13], it is still challenging to reposition the maxilla to the target position through intraoperative guidance, and to fine-tune the position of the maxilla. It is also difficult to hold the maxilla stably in the internal fixation procedures, including drilling holes and applying a plate [12]. For template-based surgery or PSI, several clinical studies have been conducted to assess the surgical accuracy and the results of these studies showed high accuracy compared to the results of surgery using traditional methods [9,10,14]. However, in the template-based surgery or PSI, a relatively long laboratory time is required to fabricate surgical tools, and there is no leeway to change maxillary positioning perioperatively, when soft tissue response is different from the preoperative prediction or there are errors in the preoperative evaluation and surgical plan [9,10,15]. In addition, excessive exposure of operation field is needed to place templates or PSI [14].

The medical robot has been applied to various surgical fields with advantages such as overcoming human hand’s limitation, minimally invasive surgery, low postoperative discomfort, and short hospital stay [16,17,18,19,20]. In orthognathic surgery, there have been several attempts to apply a robot, which have been largely carried out in two aspects: osteotomy and repositioning of the osteotomized bone segment. To our knowledge, there was only one study that introduced a robot system for maxillary osteotomies, and the investigators reported successful results in the preliminary experiments [21]. Regarding the repositioning of the bone segment, a robot arm can be effectively utilized to reposition the maxillary segment to the target position, when three-dimensional information of head position is available with the help of navigation [6]. Moreover, laboratory works for templates or implants are not necessary, therefore, perioperative change of maxillary positioning is relatively easy and comfortable by in situ modification of simulation surgery in short time. Burgner et al. [22] introduced fundamental concepts of the robot system for repositioning of the maxilla, and Wang et al. [23] proposed a system based on the cooperation of the surgical navigation and the robot arm. Previously, we also introduced a system for repositioning of the maxilla using a robot arm and image-guided navigation [24]. Despite several reports of applying the robot arm to the maxillary repositioning, the accuracy and feasibility have not been evaluated in an experimental environment that simulates the entire surgical procedures of maxillary orthognathic surgery.

In this study, we investigated the feasibility of robot-assisted maxillary repositioning in orthognathic surgery and assessed the surgical accuracy using postoperative computed tomography (CT) analysis.

## 2. Materials and Methods

The experiments in this study were performed on 12 full skull phantom models (Sawbones; Pacific Research Laboratories Inc., Vashon, WA, USA). For image-guided navigation, the initial position of the maxilla was recorded prior to the maxillary osteotomy. After Le Fort I osteotomy using a hand-held saw, the maxillary segment was repositioned to the different target positions using a robot-robot arm and navigation system. The surgical plans implemented in this study are described in Table 1, and the experiment was performed three times for each of the four surgical plans. During the maxillary repositioning, the maxilla was navigated with an optical tracking system in real time. Before the fixation of the maxilla, the achieved maxillary position relative to the surgical plan was evaluated through the navigation system. The maxillary segment was stabilized using four miniplates at the piriform aperture and zygomatic buttress on both sides. Immediately after surgery, postoperative CT images were obtained to compare the surgical outcome with the surgical plan.

### 2.1. System Implementation

**Figure 1:**
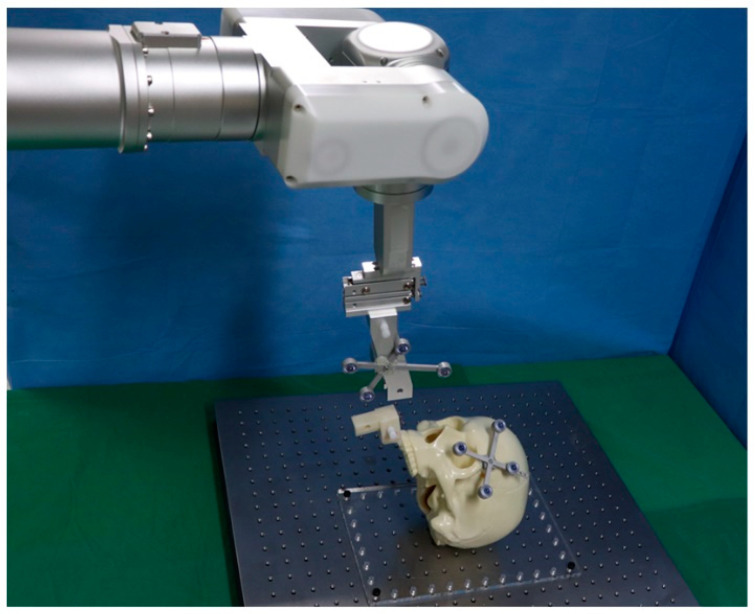
A robot arm combined with a navigation system for maxillary orthognathic surgery.

We used a robot arm with 6 degree of freedom (Cyborg-Lab, Suwon, Korea) to reposition the maxillary segment to target position (Figure 1). The robot arm is controlled by a robot motion controller provided by the manufacturer (Precise Automation, Fremont, CA, USA) and has the repeatability of ±0.15 mm and the maximum payload of 5 kg. The end-effector of robot arm can be easily coupled and detached from an occlusal splint. A tracking tool for navigation is located on the side of the end-effector to track the movement of the robot arm and the position of the maxilla. An end-effector of the robot arm has a single-axis slider which allows the surgeon to move the maxillary segment manually in the cranial-caudal direction, helping to recognize and remove bony interferences.

**Figure 2:**
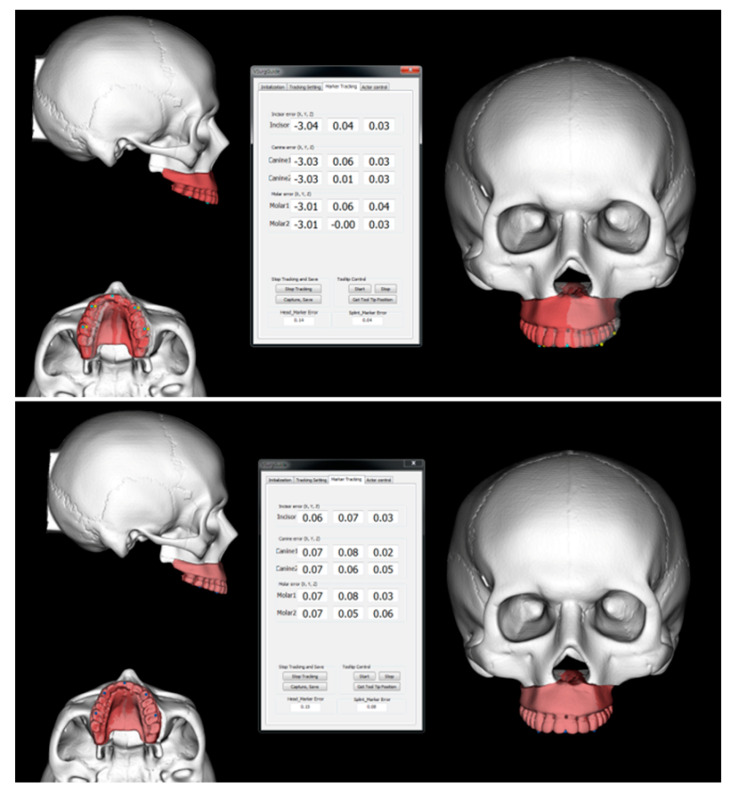
Image-guided navigation using the optical tracking system. The target and actual positions of the maxilla is visualized in real time and the discrepancy between two positions at the five dental landmarks is also provided.

Image-guided navigation is done using the optical tracking system (OTS) (POLARIS SPECTRA, Northern Digital Inc., ON, Canada) [13]. The target and actual positions of the maxilla are visualized in real time and the discrepancy between two positions at the five dental landmarks is also provided (Figure 2).

### 2.2. Experimental Procedures

**Figure 3:**
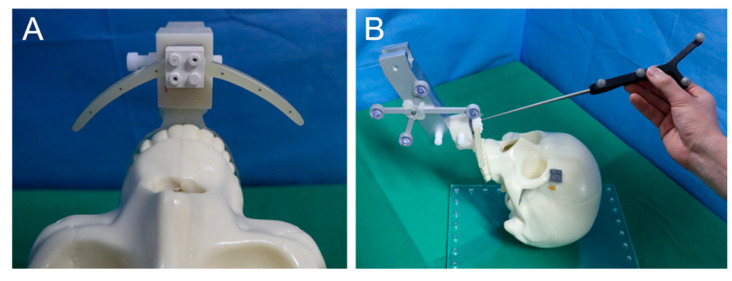
Preoperative registration for matching the physical space and the CT image space. (**A**) Registration body; (**B**) acquisition of the physical positions of spheres on the registration body related to the tracking tool on the end-effector.

Preoperative CT images are obtained after the occlusal splint is attached to the maxillary teeth. To match the CT space and the physical space, the registration body was connected to the splint during CT scan (Figure 3A). After segmentation of virtual 3D skull model using CT images, optical scan data of the maxillary dentition were registered with the skull model using the inspection program (GOM Inspect; Gom mbH, Braunschiweig, Germany). Using the 3D simulation program (Mimics 17.0, Materialise, Leuven, Belgium), a Le Fort I osteotomy was performed on the skull model and the maxilla was moved to the target position according to the surgical plan. Finally, both the maxillary segment at the preoperative position and that at the target position were exported as stereolithography interface format (STL) files.

**Figure 4:**
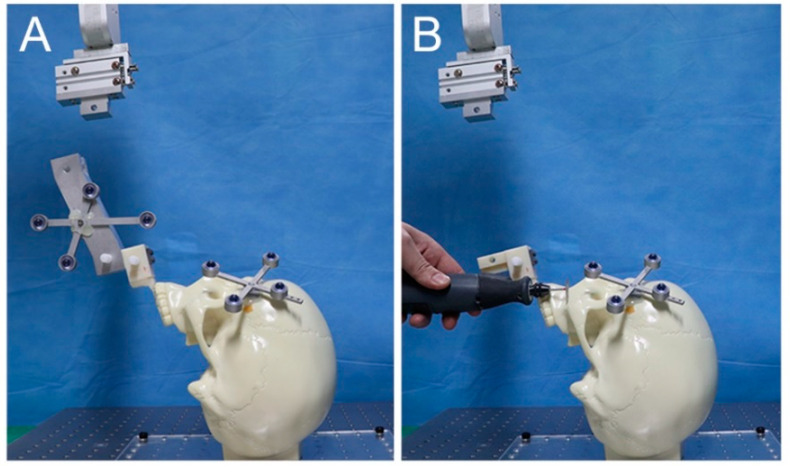
Initial intraoperative registration of the preoperative maxillary position (**A**) and Le Fort I osteotomy (**B**).

**Figure 5:**
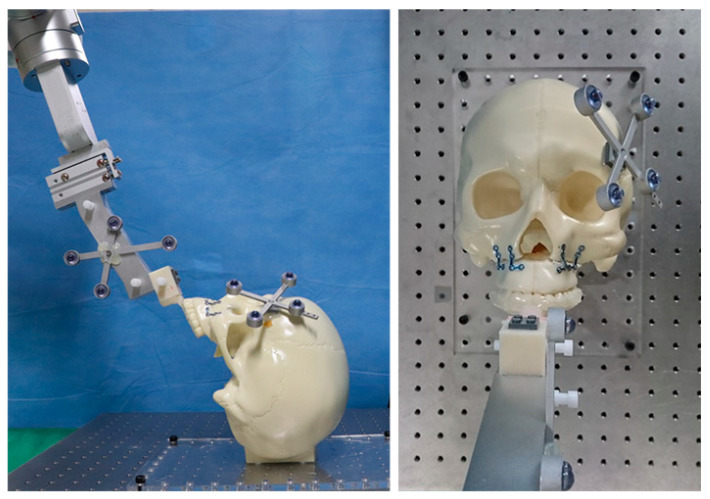
Stabilization of the repositioned maxillary segment.

To match the CT image space and the physical space, the physical positions of spheres on the registration body related to the tracking tool on the end-effector were obtained using a wireless tracking tool tip (Northern Digital Inc., ON, Canada) (Figure 3B). Then, they were registered with their corresponding spheres on the CT images. After initial intraoperative registration was performed to record the preoperative position of the maxilla in physical space, a standard Le Fort I osteotomy was performed on the phantom skull model (Figure 4). The maxillary segment was attached to the end-effector of the robot arm using the occlusal splint. The maxilla was moved to the target position using the image-guided navigation and robot arm using the robot motion controller. The bony interferences occurring during the repositioning of the maxillary segment are recognized and completely removed. After confirming the position of the repositioned maxilla by the navigation, the maxilla was stabilized using L-shaped miniplates at the pyriform aperture and zygomatic buttress (Figure 5). During osteosynthesis, the maxilla was held in the target position by the robot arm. Immediately after surgery, CT images were obtained to compare the surgical plan and the final surgical outcome.

### 2.3. Accuracy Evaluation

**Figure 6:**
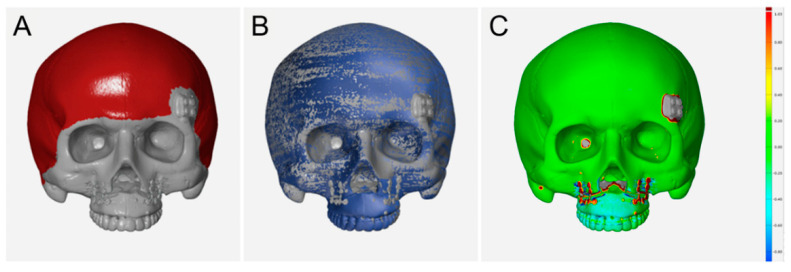
Superimposition of the postoperative 3D skull model registered with scanned dentition with the virtual surgical outcome. (**A**) Selection of the outer cortex of the cranium for local best-fit registration; (**B**) after superimposition of the two skull models; (**C**) surface comparison using a colored map.

**Figure 7:**
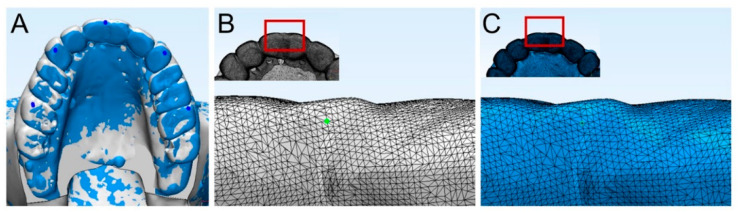
Comparison of the virtual surgical outcome and postoperative skull models. (**A**) After superimposition of the two models based on the local-best fit method; (**B**) selection of the landmark in the virtual surgical outcome using triangular nodes on the surface of teeth; (**C**) selection of the same landmark on the postoperative model using triangular nodes.

First, the maxilla was repositioned to the target position using a robot arm and a navigation system, and then the amount of deviation compared to the surgical plan was obtained through navigation. To evaluate the displacement of the maxilla, which occurred during fixation while the robot arm was holding the maxilla, the maxillary position before and after fixation was obtained, and the displacement was calculated. The final surgical accuracy was assessed by comparing the 3D skull model after virtual surgery with the 3D skull model generated from the postoperative CT images. For the postoperative skull model, registration of optical scan data of upper teeth was performed due to the limitation of resolution in CT images, as done in preoperative virtual surgery, with the same scan data. This process allowed for the selection of exactly the same dental landmarks, and the accurate measurement of the positional error at the landmarks between the planned and actual positions. Then, postoperative skull models registered with scanned dentition were superimposed with the virtual surgical outcome (Figure 6). Superimposition was done using the best fit method in selected areas, where there were no changes before and after surgery, such as the outer cortex of the cranium. The quality of superimposition was confirmed with surface comparison using a colored map and mean distance. After superimposition between two 3D models, both planned and postoperative skull models were imported into the 3D CAD software (3-Matic, Materialise NV, Leuven, Belgium). The five landmarks points of the maxilla, including the midpoint of the incisal edge of both central incisors, both canine, and the mesio-buccal cusp of both upper first molars, were digitized on both the planned and postoperative models using triangular nodes on the surface of the teeth (Figure 7). As identical scan data of dentition were used for registration of both preoperative and postoperative skull models, exactly the same triangle vertex could be selected in both. The differences in x-, y-, and z-coordinates between the planned and postoperative results were calculated.

### 2.4. Statistical Analysis

Statistical analysis was performed using the SPSS software version 25.0 (SPSS Inc., Chicago, IL, USA). To assess the significance of differences between the paired data, the Wilcoxon signed-rank test was performed. Subsequently, the mean difference between the planned and the actual maxillary position was described as the mean value, standard deviation, 95% confidence interval of the difference, and 95% limits of agreement, with a Bland–Altman plot.

## 3. Results

### 3.1. Intraoperative Evaluation of the Discrepancy between the Planned and Actual Postoperative Maxillary Position Using Navigation

Using the robot arm and navigation system, the maxilla was repositioned to be as close as possible to the target position with mean absolute deviations of 0.10 ± 0.12 mm, 0.08 ± 0.07 mm, and 0.10 ± 0.10 mm in medio-lateral, antero-posterior, and supero-inferior directions, respectively, from the target position and the mean root mean square deviation (RMSD) was 0.19 ± 0.14 mm (Table 2). During the stabilization of the maxilla with miniplates, the maxillary segment exhibited displacements of 0.22 ± 0.19 mm, 0.18 ± 0.12 mm, 0.18 ± 0.12 mm, and 0.37 ± 0.20 mm in medio-lateral, antero-posterior, and supero-inferior directions, and in three-dimension, respectively, compared to the pre-stabilized maxilla (Table 3). When the fixation of the maxilla was completed, mean absolute deviations compared to the target position were 0.16 ± 0.13 mm, 0.18 ± 0.15 mm, and 0.20 ± 0.16 mm in medio-lateral, antero-posterior, and supero-inferior directions, respectively, and the mean RMSD was 0.35 ± 0.21 mm. Although there were statistically significant increases in the absolute deviations of the maxilla during fixation (medio-lateral, 0.06 mm, *p* = 0.020; antero-posterior, 0.10 mm, *p* < 0.001; supero-inferior, 0.10 mm, *p* < 0.001; RMSD, 0.15 mm, *p* = 0.012), the mean amount of increase was not clinically significant.

### 3.2. Postoperative Evaluation of the Discrepancy between the Planned and Actual Postoperative Maxillary Position Using CT Data

The quantitative quality of superimposition between the 3D skull models after the virtual surgery and the actual surgery was confirmed by a deviation in the reference surface, that was 0.05 ± 0.01 mm on average (range, 0.04 mm to 0.08 mm).

The mean absolute discrepancy between the planned and actual postoperative positions of the maxilla was 0.42 ± 0.35 mm (range, 0.01 to 1.07), 0.37 ± 0.25 mm (range, 0.05 to 1.05), and 0.38 ± 0.32 mm (range, 0.00 to 1.41) in medio-lateral, antero-posterior, and supero-inferior directions, respectively, and the mean RMSD was 0.81 ± 0.31 mm (range, 0.35 to 1.18) (Table 4). More details regarding the planned and actual movements of the maxilla are described in Supplementary Table S1. The mean 3D distance between the planned and actual postoperative maxilla was 0.79 ± 0.35 mm (range, 0.19 to 1.62). Depending on the region of the maxilla, central incisor, right and left canine, and right and left upper first molar exhibited 3D distance between the planned and actual postoperative maxilla by 0.76 ± 0.42 mm (range, 0.32 to 1.62), 0.85 ± 0.38 mm (0.39 to 1.58), 0.70 ± 0.31 mm (0.26 to 1.27), 0.91 ± 0.33 mm (0.44 to 1.48), and 0.72 ± 0.30 (0.19 to 1.06), respectively.

**Figure 8:**
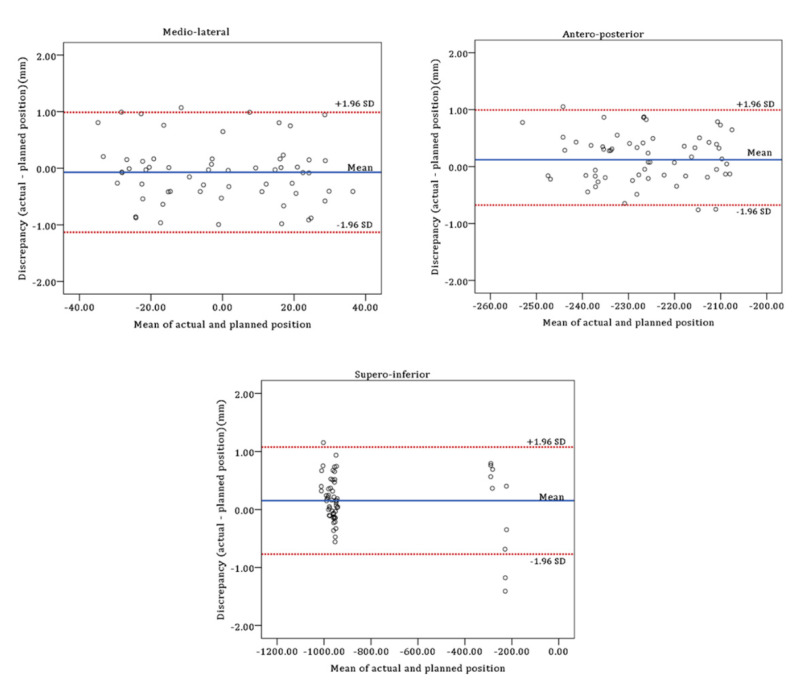
Bland-Altman plot of the differences in the medio-lateral, antero-posterior, and supero-inferior directions between the planned and actual postoperative maxillary positions.

The 95% confidence intervals (CI) for the mean positional difference between the planned and the actual postoperative maxilla were −0.21 mm to 0.07 mm, 0.05 mm to 0.27 mm, and 0.03 mm to 0.28 mm in medio-lateral, antero-posterior, and supero-inferior directions, respectively (Table 5). The 95% of limits of agreement (LOA) between the planned and the actual postoperative maxillary position were −1.13 mm to 0.99 mm, −0.68 mm to 0.99 mm, and −0.77 mm to 1.08 mm in the medio-lateral, antero-posterior, and supero-inferior directions, respectively (Figure 8).

## 4. Discussion

Surgical navigation has been widely applied to maxillofacial surgery, and orthognathic surgery is one of the areas being actively studied [25]. Surgical navigation can help in real-time accurate identification of important anatomical structures, transfer of surgical plans, and verification of surgical results [26]. In this study, we combined a robot arm with a surgical navigation, and evaluated the accuracy of surgical outcome when performing orthognathic surgery using this system in the laboratory set-up. The results of this study demonstrate that the robot arm combined with the navigation system may enable accurate maxillary orthognathic surgery by tracking the position of the maxilla during the entire surgical procedures and moving the maxilla to the target position with fine adjustment.

Previously, we reported an orthognathic surgery system using an image-guided navigation using optical tracking or electromagnetic tracking, and the navigation-based orthognathic surgery could help to accurately reposition the maxilla in the target position [13,27]. Mazzoni et al. [28] reported that preoperative surgical plan reproducibility was 86.5% with the help of intraoperative navigation based on the optical tracking, and 80% without the navigation. In contrast, in the study by Berger et al. [12], there was no difference in the accuracy of the maxillary repositioning between the electromagnetic-navigated maxillary repositioning and conventional splint-based repositioning. Although intraoperative navigation can help to accurately move the maxilla to the planned position, difficulties in maintaining the repositioned maxilla stably during the subsequent surgical procedures including fixation have been raised [12,29]. To solve the inaccuracy problem that may occur during the fixation, the loose maxilla was held manually and temporarily supported with bone wax in the previous study [12]. In our study, a robot arm was combined with the surgical navigation to maintain the maxillary segment in the target position during the fixation period, and the maxilla exhibited a displacement of 0.22 mm, 0.18 mm, and 0.18 mm in medio-lateral, antero-posterior, and supero-inferior directions, respectively.

In the present study, the postoperative maxilla exhibited mean absolute deviations of 0.42 mm in medio-lateral direction, 0.37 mm in antero-posterior direction, and 0.38 mm in supero-inferior direction, compared with the established surgical plan. The mean RMSD was 0.8 mm. The results in the present study are comparable to the surgical accuracy of template-based surgery or PSI, which has recently been increasing in clinical application with high accuracy and decreased operation time. Heufelder et al. [9] reported that the median deviation between the planned and postoperative maxillary position was 0.30 mm in left/right positioning, 0.33 mm in up/down positioning, and 0.7 mm in anterior-posterior positioning after waferless maxillary positioning using customized surgical guides and PSI. In the comparison of maxillary repositioning between PSI and interocclusal splint by Ruckschloss et al. [10], PSI group showed the discrepancies of 0.51 mm in left/right positioning, 0.39 mm in anterior/posterior positioning, and 0.44 mm in up/down positioning. Recently, Wong et al. [14] also assessed the accuracy of maxillary repositioning using customized titanium guides and PSI, and they reported the overall root mean error of 0.38 mm in the transverse dimension, 0.64 mm in the anteroposterior dimension, and 0.55 mm in the vertical dimension.

While successful results of the surgical devices, such as intermaxillary splints, customized surgical guides or patient-specific implants, used in the past or present have been reported, a lack of flexibility in unexpected events during surgery has been raised as one of the drawbacks [29,30]. The surgical devices require a considerable preparation period before surgery for design and fabrication. Thus, it is difficult to change the surgical plans recorded on the surgical plan when preoperative diagnosis or planning is inappropriate. In addition, the mal-produced surgical devices can also cause difficulties for surgeons during surgery. In the clinical study where the surgical experience of patient-specific devices was reported in 30 patients, changing of the screw holes and additional bending of the metal plane was required intraoperatively in one-third of them [30]. For 3 patients in that study, patients-specific devices could not be used. In contrast, the robot arm combined with the surgical navigation can provide flexibility in surgical plan during surgery. In the maxillary repositioning using a robot arm, a robot arm continuously tracks the current position of the maxilla through the navigation, thus it is possible to move the maxilla from the originally planned position according to the revised plan.

Robot arm can be introduced into orthognathic surgery in various ways depending on the level of autonomy of the robot. In this study, the robot arm was passively manipulated by a surgeon with the help of continuous navigation tracking, and the robot arm performed the tasks of precisely controlling the position of the maxilla. Of course, ultimately, fully autonomous maxillary surgery would be technically feasible, however this method may not be clinically desirable unless reliable safety devices that can completely prevent robot malfunctions are developed [25]. In addition, in the automated method, the accuracy of surgery may be significantly affected by the registration errors. Another limitation of fully autonomous orthognathic surgery may be that it is difficult to accurately recognize bony interference and remove it properly using a robot arm. To recognize bony interference that prevents maxillary repositioning to the target position, repetition of a series of processes of recognizing and removing bone interference while moving the maxilla to the target position is required. Through this repetitive process, the location of bony interference can be confirmed with the visual or tactile sense. Since the robot arm lacks tactile perception and proprioception, it may be difficult to effectively recognize bony interference, and the process of repeatedly operating the robot arm to move the maxilla close to or far from the target position may be time-consuming and cumbersome [17]. In the robot arm used in this study, a slider was attached to the end-effector to help recognize and remove bony interference. By moving the slider in the cranial direction, the bony interference could be felt and recognized. When removing bony interference, the maxilla was moved caudally using a slider to obtain a space for access of rotary cutting instrument.

Analysis using 3D CT data is becoming a common method for analyzing surgical results in the oral and maxillofacial region. However, CT analysis has several inherent limitations. At first, as CT data are usually a set of two-dimensional cross-sectional images with slice thickness usually ranging from 0.3 mm to 1.5 mm, the reconstructed 3D skull model created using these cross-sectional images can be different from the actual skull model. Furthermore, it is difficult to digitize the landmark in the exact same location on the two skull models generated from different CT data in one patient. One of the ways to overcome the inherent limitations of CT analysis is to use dental markers such as orthodontic brackets attached to the maxillary teeth. The dental markers can also be used in place of the registration body that was used during the preoperative registration for matching the physical space and CT image [31]. In our study, to minimize these errors in the CT evaluation, high-resolution optical scan surface data are registered with both the preoperative and postoperative 3D skull models in this study. Registration of the same scan data into preoperative and postoperative 3D skull models allowed the identification of the same surface triangular structures in dentition in both the 3D models, thus making it possible to select the exact same landmarks for evaluation.

In conclusion, we could confirm the potential of the maxillary orthognathic surgery using the system that combined robot arm and surgical navigation. However, the proposed protocol using robot arm and navigation has several limitations. First, additional cost for robot arm and navigation system, and the space in the operating room to accommodate the robot system are required. In addition, preparation time in and out of the operating room, including software-related tasks to import the surgical plan into the navigation system and maneuvering the robot arm using the robot motion controller, is required. Since the system in the present study is in the laboratory stage, to apply the system clinically, it is also necessary to refine the equipment to suit the clinical setting. The patient’s head moves in actual surgery, so it is necessary to develop a device that can fix the head during surgery or to improve the system to compensate for the change in the position of the head. In addition, development of an end-effector and software that can visualize the degree and location of resistance when the robot arm moved the maxilla may be necessary to overcome the limitations of a robot arm that cannot perform tactile sensation and secure complete safety.

## Figures and Tables

**Table 1:** Description of different surgical plans implemented in this study.

Type of Movement	Direction and Amount of Movement	Number of Experiments
Bodily shift	Advancement by 3 mm and downward by 2 mm	3
	Bodily shift to the right by 3 mm	3
Rotation	Cant correction by 4 mm (#16: upward by 2 mm, #26: downward by 2 mm)	3
	Posterior impaction (#16 and #26: upward by 3 mm)	3

**Table 2:** Mean absolute deviation based on intraoperative navigation before and after maxillary fixation.

Land- Marks	Medio-Lateral			Antero-Posterior			Supero-Inferior		
	Before Fixation (mm)	After Fixation (mm)	*p* Value *	Before Fixation (mm)	After Fixation (mm)	*p* Value *	Before Fixation (mm)	After Fixation (mm)	*p* Value *
#11	0.11 ± 0.14	0.14 ± 0.14	0.432	0.05 ± 0.05	0.17 ± 0.15	0.011	0.13 ± 0.15	0.22 ± 0.22	0.071
#13	0.11 ± 0.13	0.15 ± 0.13	0.530	0.08 ± 0.05	0.23 ± 0.18	0.003	0.12 ± 0.10	0.23 ± 0.17	0.041
#23	0.11 ± 0.13	0.15 ± 0.13	0.530	0.07 ± 0.05	0.12 ± 0.12	0.209	0.09 ± 0.11	0.18 ± 0.18	0.023
#16	0.10 ± 0.11	0.18 ± 0.14	0.084	0.08 ± 0.06	0.24 ± 0.18	0.002	0.08 ± 0.05	0.19 ± 0.11	0.021
#26	0.10 ± 0.10	0.18 ± 0.14	0.084	0.11 ± 0.11	0.14 ± 0.11	0.432	0.09 ± 0.10	0.18 ± 0.11	0.037
Total	0.10 ± 0.12	0.16 ± 0.13	0.020	0.08 ± 0.07	0.18 ± 0.15	<0.001	0.10 ± 0.10	0.20 ± 0.16	<0.001

Data are presented as mean ± standard deviation. * Wilcoxon signed-rank test.

**Table 3:** Displacement of the mobilized maxilla during fixation based on intraoperative navigation.

Land-Marks	Medio-Lateral		Antero-Posterior		Supero-Inferior	
	Mean ± SD (mm)	*p* Value *	Mean ± SD (mm)	*p* Value *	Mean ± SD (mm)	*p* Value *
#11	0.22 ± 0.19	0.002	0.18 ± 0.12	0.328	0.16 ± 0.12	0.307
#13	0.23 ± 0.19	0.002	0.19 ± 0.14	0.326	0.19 ± 0.10	0.530
#23	0.20 ± 0.19	0.002	0.16 ± 0.09	0.388	0.15 ± 0.13	0.195
#16	0.23 ± 0.20	0.002	0.21 ± 0.14	0.213	0.22 ± 0.11	0.656
#26	0.23 ± 0.20	0.002	0.15 ± 0.08	0.724	0.17 ± 0.13	0.286
Total	0.22 ± 0.19	<0.001	0.18 ± 0.12	0.303	0.18 ± 0.12	0.426

Data are presented as mean ± standard deviation. * Wilcoxon signed-rank test.

**Table 4:** Mean absolute deviation between the planned and actual postoperative maxillary positions.

Land-Marks	Medio-Lateral			Antero-Posterior			Supero-Inferior		
	Mean ± SD (mm)	Min (mm)	Max (mm)	Mean ± SD (mm)	Min (mm)	Max (mm)	Mean ± SD (mm)	Min (mm)	Max (mm)
#11	0.39 ± 0.36	0.03	1.07	0.39 ± 0.22	0.16	0.87	0.38 ± 0.38	0.10	1.41
#13	0.39 ± 0.37	0.01	0.99	0.47 ± 0.27	0.15	1.05	0.42 ± 0.34	0.02	1.18
#23	0.40 ± 0.37	0.01	0.99	0.30 ± 0.23	0.05	0.83	0.31 ± 0.25	0.03	0.79
#16	0.45 ± 0.35	0.07	0.96	0.48 ± 0.28	0.15	0.87	0.44 ± 0.35	0.00	1.15
#26	0.46 ± 0.35	0.08	0.95	0.24 ± 0.20	0.05	0.73	0.34 ± 0.28	0.03	0.75
Total	0.42 ± 0.35	0.01	1.07	0.37 ± 0.25	0.05	1.05	0.38 ± 0.32	0.00	1.41

Abbreviations: SD, standard deviation; Min, minimum; Max, maximum.

**Table 5:** Mean and standard deviation of discrepancy, 95% confidence intervals and limits of agreement for mean discrepancy.

			Limits of Agreement (95% Confidence Interval)	
	Mean ± SD (mm)	95% CI for Mean Discrepancy	Lower Limit	Upper Limit
*Signed difference*				
Medio-lateral	−0.07 ± 0.54	−0.21 to 0.07	−1.13 (−1.37 to −0.89)	0.99 (0.75 to 1.23)
Antero-posterior	0.16 ± 0.43	0.05 to 0.27	−0.68 (−0.87 to −0.49)	0.99 (0.80 to 1.18)
Supero-inferior	0.15 ± 0.47	0.03 to 0.28	−0.77 (−0.98 to −0.56)	1.08 (0.87 to 1.29)
*Absolute difference*				
Medio-lateral	0.42 ± 0.35	0.33 to 0.51	−0.27 (−0.42 to −0.11)	1.10 (0.94 to 1.25)
Antero-posterior	0.37 ± 0.25	0.31 to 0.44	−0.12 (−0.24 to −0.01)	0.87 (0.76 to 0.98)
Supero-inferior	0.38 ± 0.32	0.30 to 0.46	−0.24 (−0.38 to −0.10)	1.00 (0.86 to 1.14)

Abbreviations: SD, standard deviation; CI, confidence intervals.

## Data Availability

The data presented in this article are available within this article.

## Supplementary Materials

The following are available online at https://www.mdpi.com/article/10.3390/jcm10122596/s1. Table S1: The planned and actual movements of the maxilla depending on each surgical plan.
