# Supplementary material for: Robot-Assisted Maxillary Positioning in Orthognathic Surgery: A Feasibility and Accuracy Evaluation
Source: J Clin Med. 2021 Jun 11;10(12):2596. doi: 10.3390/jcm10122596 (PMC8231103; doi:10.3390/jcm10122596)
Supplement: Supplementary file 1 [file jcm-10-02596-s001.zip › jcm-1205275-supplementary.pdf]

Table S1. The planned and actual movements of the maxilla depending on each surgical plan.

	Planned Movement (mm)			Actual Movement (mm)		
	x	y	z	x	y	z
**Bodily shift**						
Advancement by 3 mm and downward by 2mm						
#11	0.00 ± 0.00	−3.00 ± 0.00	−2.00 ± 0.00	0.30 ± 0.67	−2.72 ± 0.50	−2.26 ± 1.09
#13	0.00 ± 0.00	−3.00 ± 0.00	−2.00 ± 0.00	0.32 ± 0.58	−2.67 ± 0.70	−2.32 ± 0.87
#23	0.00 ± 0.00	−3.00 ± 0.00	−2.00 ± 0.00	0.32 ± 0.58	−2.76 ± 0.29	−1.98 ± 0.74
#16	0.00 ± 0.00	−3.00 ± 0.00	−2.00 ± 0.00	0.31 ± 0.45	−2.70 ± 0.61	−2.18 ± 0.48
#26	0.00 ± 0.00	−3.00 ± 0.00	−2.00 ± 0.00	0.32 ± 0.45	−2.84 ± 0.15	−1.68 ± 0.42
Bodily shift to the right by 3 mm						
#11	−3.00 ± 0.00	0.00 ± 0.00	0.00 ± 0.00	−3.02 ± 0.24	0.37 ± 0.53	0.21 ± 0.27
#13	−3.00 ± 0.00	0.00 ± 0.00	0.00 ± 0.00	−3.03 ± 0.23	0.34 ± 0.50	0.22 ± 0.44
#23	−3.00 ± 0.00	0.00 ± 0.00	0.00 ± 0.00	−3.03 ± 0.23	0.39 ± 0.54	0.25 ± 0.33
#16	−3.00 ± 0.00	0.00 ± 0.00	0.00 ± 0.00	−3.06 ± 0.21	0.30 ± 0.43	0.28 ± 0.71
#26	−3.00 ± 0.00	0.00 ± 0.00	0.00 ± 0.00	−3.07 ± 0.21	0.37 ± 0.45	0.31 ± 0.57
**Rotation**						
Cant correction by 4mm						
#11	0.00 ± 0.00	0.00 ± 0.00	0.02 ± 0.05	−0.58 ± 0.36	−0.02 ± 0.34	0.33 ± 0.21
#13	0.01 ± 0.05	0.00 ± 0.00	1.38 ± 0.05	−0.59 ± 0.30	−0.08 ± 0.45	1.85 ± 0.39
#23	−0.02 ± 0.07	0.00 ± 0.00	−1.37 ± 0.04	−0.64 ± 0.30	0.04 ± 0.26	−1.08 ± 0.15
#16	0.46 ± 0.18	0.00 ± 0.00	1.95 ± 0.04	−0.15 ± 0.16	−0.16 ± 0.59	2.59 ± 0.60
#26	0.47 ± 0.19	0.00 ± 0.00	−2.01 ± 0.05	−0.16 ± 0.20	0.01 ± 0.27	−1.64 ± 0.34
Posterior impaction by 3 mm						
#11	0.00 ± 0.00	0.00 ± 0.00	0.00 ± 0.00	0.03 ± 0.59	0.06 ± 0.44	0.00 ± 0.21
#13	0.00 ± 0.00	−0.17 ± 0.16	1.00 ± 0.03	0.04 ± 0.70	−0.08 ± 0.79	0.98 ± 0.16
#23	0.00 ± 0.00	−0.31 ± 0.10	1.14 ± 0.07	0.04 ± 0.71	−0.28 ± 0.34	1.15 ± 0.09
#16	0.00 ± 0.00	−1.07 ± 0.18	3.00 ± 0.00	0.07 ± 0.92	−0.98 ± 0.93	3.12 ± 0.10
#26	0.00 ± 0.00	−1.38 ± 0.19	3.16 ± 0.21	0.06 ± 0.93	−1.39 ± 0.35	3.19 ± 0.19

Data are presented as mean ± standard deviation.
